# Calcified fibrous tumor in the stomach: a rare case of gastric submucosal tumor treated with endoscopic submucosal excavation

**DOI:** 10.1055/a-2197-9341

**Published:** 2023-11-21

**Authors:** Liansong Ye, Meiting Liang, Shuai Bai, Xinyue Zhou, Ou Chen, Bing Hu, Yi Mou

**Affiliations:** 1 34753 Department of Gastroenterology and Hepatology, West China Hospital, Sichuan University, Chengdu, China

A 39-year-old woman presented with a small submucosal tumor in the posterior wall of gastric lower body ( Fig. 1 ). She reported nonspecific gastrointestinal symptoms such as recurrent epigastric pain and abdominal distension for 4 years. There was no significant medical history. Physical examination revealed no obvious abnormalities. Endoscopic ultrasonography showed the hypoechoic tumor with a clear boundary and an endoluminal growth pattern, which originated from the muscularis propria ( Fig. 2 ). Enhanced computed tomography confirmed the tumor in the gastric body ( Fig. 3 ).

**Fig. 1:**
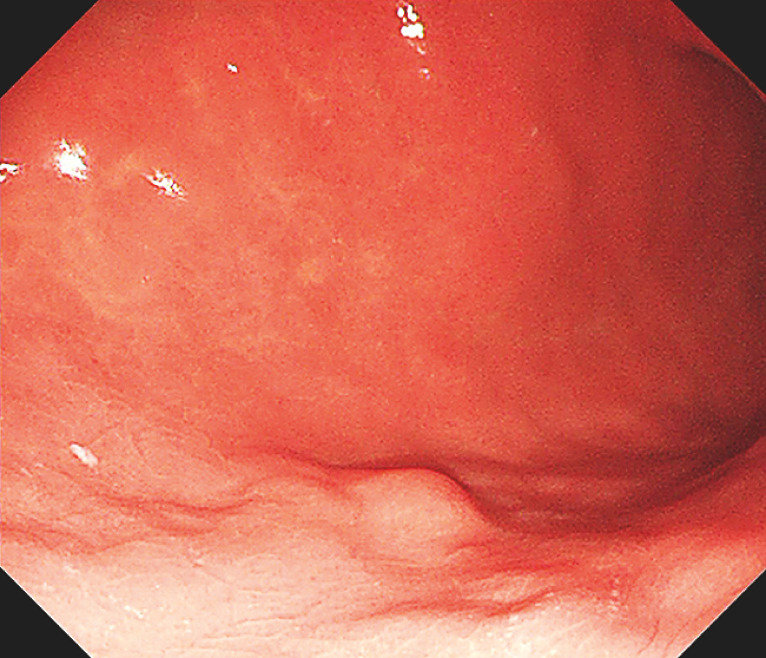
Upper endoscopy revealed a small submucosal tumor in the posterior wall of the gastric lower body.

**Fig. 2:**
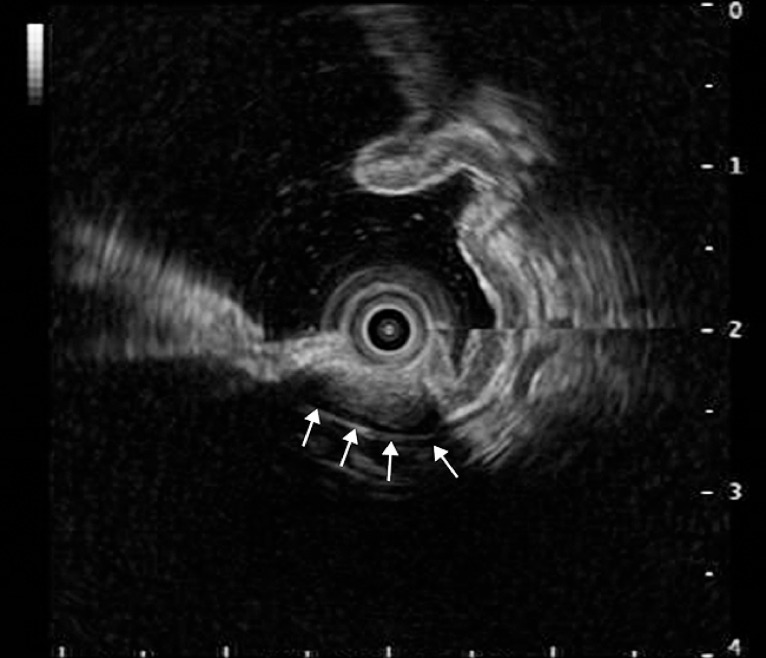
Endoscopic ultrasonography showed the hypoechoic tumor with a clear boundary and an endoluminal growth pattern, which originated from the muscularis propria (arrows).

**Fig. 3:**
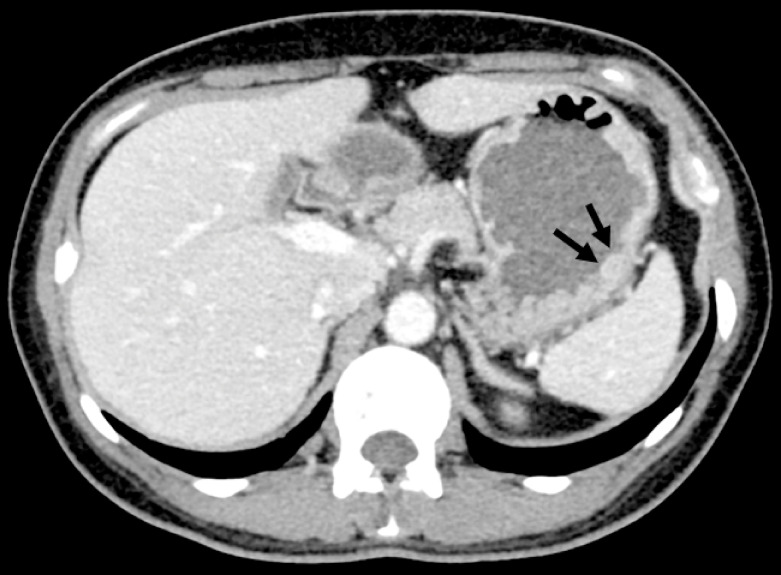
Enhanced computed tomography confirmed the lesion with an endoluminal growth pattern in the gastric body (arrows).

Following a strong request from the patient, we performed endoscopic submucosal excavation (ESE) using a dual knife ( Video 1 ). Complete resection of the tumor was achieved ( Fig. 4 ). Pathology showed a collagenous nodule under gastric mucosa, and the vertical margin was normal muscularis propria ( Fig. 5 ). Immunohistochemistry revealed that the tumor was positive for CD34, but negative for CD117, DOG-1, Desmin, SMA, S100, and IgG4. Therefore, the diagnosis of gastric calcified fibrous tumor (CFT) was made.

**Fig. 4:**
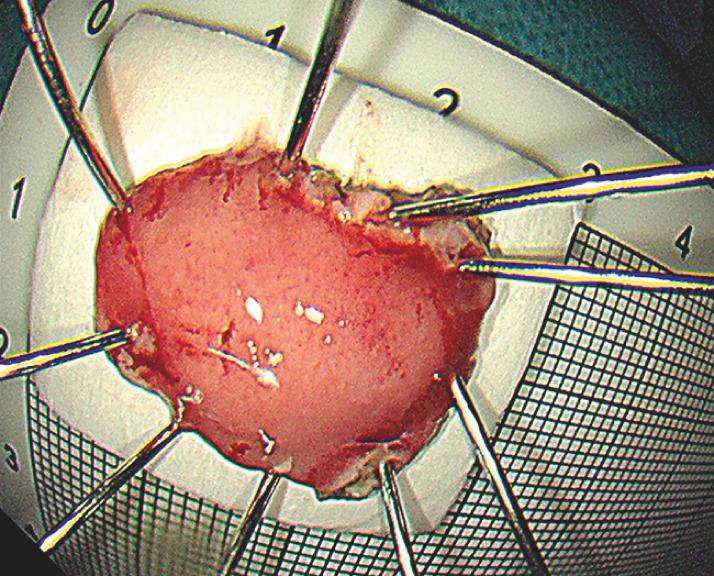
The resected specimen.

**Fig. 5:**
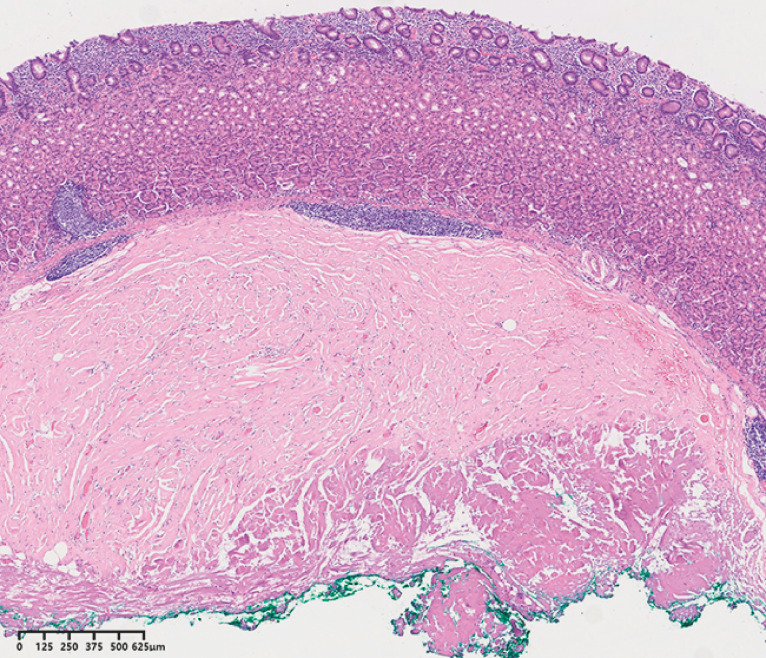
Hematoxylin and eosin staining (×30) showed a collagenous nodule under the gastric mucosa, with invasion of the muscularis propria, suggesting gastric calcified fibrous tumor.

Endoscopic submucosal excavation of a calcified fibrous tumor in the stomach.Video 1

The patient kept fasting for 24 hours and began to drink after 1 day. She received proton pump inhibitors and antibiotic prophylaxis. Her recovery was uneventful, and she was discharged after 2 days.

Gastric CFT is an extremely rare mesenchymal benign tumor with unclear pathogenesis, which is easily confused with common spindle cell lesions of the gastrointestinal tract, especially leiomyoma and gastrointestinal stromal tumors 1 . The clinical implication of gastric CFT is not clear, and surgical resection remains the main treatment 2 . Our experience suggests that ESE can be an alternative for resection of gastric CFT.

Endoscopy_UCTN_Code_CCL_1AB_2AD_3AB

## Notes

Endoscopy E-Videos https://eref.thieme.de/e-videos
*E-Videos* is an open access online section of the journal *Endoscopy* , reporting on interesting cases and new techniques in gastroenterological endoscopy. All papers include a high-quality video and are published with a Creative Commons CC-BY license. Endoscopy E-Videos qualify for HINARI discounts and waivers and eligibility is automatically checked during the submission process. We grant 100% waivers to articles whose corresponding authors are based in Group A countries and 50% waivers to those who are based in Group B countries as classified by Research4Life (see: https://www.research4life.org/access/eligibility/ ). This section has its own submission website at https://mc.manuscriptcentral.com/e-videos .
